# Gut microbiota in adults with moyamoya disease: characteristics and biomarker identification

**DOI:** 10.3389/fcimb.2023.1252681

**Published:** 2023-10-17

**Authors:** Xiaofan Yu, Peicong Ge, Yuanren Zhai, Wei Liu, Qian Zhang, Xun Ye, Xingju Liu, Rong Wang, Yan Zhang, Jizong Zhao, Dong Zhang

**Affiliations:** ^1^ Department of Neurosurgery, Beijing Tiantan Hospital, Capital Medical University, Beijing, China; ^2^ China National Clinical Research Center for Neurological Diseases, Beijing, China; ^3^ Center of Stroke, Beijing Institute for Brain Disorders, Beijing, China; ^4^ Beijing Key Laboratory of Translational Medicine for Cerebrovascular Disease, Beijing, China; ^5^ Beijing Translational Engineering Center for 3D Printer in Clinical Neuroscience, Beijing, China; ^6^ Savaid Medical School, University of Chinese Academy of Sciences, Beijing, China; ^7^ Department of Neurosurgery, Beijing Hospital, Beijing, China

**Keywords:** gut microbiota, characteristics, biomarker, moyamoya disease, adults

## Abstract

**Background and purpose:**

When it comes to the onset of moyamoya disease (MMD), environmental variables are crucial. Furthermore, there is confusion about the relationship between the gut microbiome, an environmental variable, and MMD. Consequently, to identify the particular bacteria that cause MMD, we examined the gut microbiome of MMD individuals and healthy controls (HC).

**Methods:**

A prospective case-control investigation was performed from June 2021 to May 2022. The fecal samples of patients with MMD and HC were obtained. Typically, 16S rRNA sequencing was employed to examine their gut microbiota. The QIIME and R softwares were used to examine the data. The linear discriminant analysis effect size analysis was used to determine biomarkers. Multivariate analysis by linear models (MaAsLin)2 were used to find associations between microbiome data and clinical variables. Model performance was assessed using the receiver operating characteristic curve and the decision curve analysis.

**Results:**

This investigation involved a total of 60 MMD patients and 60 HC. The MMD group’s Shannon and Chao 1 indices were substantially lower than those of the HC cohort. β-diversity was significantly different in the weighted UniFrac distances. At the phylum level, the relative abundance of *Fusobacteriota*/*Actinobacteria* was significantly higher/lower in the MMD group than that in the HC group. By MaAsLin2 analysis, the relative abundance of the 2 genera, *Lachnoclostridium* and *Fusobacterium*, increased in the MMD group, while the relative abundance of the 2 genera, *Bifidobacterium* and *Enterobacter* decreased in the MMD group. A predictive model was constructed by using these 4 genera. The area under the receiver operating characteristic curve was 0.921. The decision curve analysis indicated that the model had usefulness in clinical practice.

**Conclusions:**

The gut microbiota was altered in individuals with MMD, and was characterized by increased abundance of *Lachnoclostridium* and *Fusobacterium* and decreased abundance of *Bifidobacterium* and *Enterobacter*. These 4 genera could be used as biomarkers and predictors in clinical practice.

## Introduction

The development of intracranial carotid artery stenosis and an irregular vascular network in the brain are characteristics of moyamoya disease (MMD), an uncommon kind of cerebrovascular disorder (Suzuki and Takaku, 1969; Scott and Smith, 2009). MMD is a rare cerebrovascular condition, yet despite this, it is the most common reason for stroke in children in East Asian nations (Kuriyama et al., 2008; Kim et al., 2016). There are two primary phenotypes of MMD in these nations, according to the presentation of MMD: hemorrhagic and ischemic kind (Liu et al., 2015).

Though the epidemiology, clinical features, and treatment of MMD have been thoroughly studied (Liu et al., 2015; Acker et al., 2018), little is known about its etiology and progression. Several studies have shown that MMD may be associated with immune, inflammatory, and genetic causes (Masuda et al., 1993; Fujimura et al., 2014; Bang et al., 2016). A pathological study has revealed the existence of growing vascular smooth muscle cells accompanied with inflammatory cells in the intima of stenotic or obstructive intracranial arterial lesions (Masuda et al., 1993). Another prospective study has indicated significantly elevated circulating Treg and Th17 cells in subjects with MMD compared to healthy controls (HC) (Weng et al., 2017). A complicated procedure involving hereditary and environmental variables is likely what causes chronic inflammation (Renz et al., 2011). A significant correlation between RNF213 p.R4810K variant and MMD has also been identified (Kamada et al., 2011; Liu et al., 2011). However, the penetrance of RNF213 p.R4810K is lower than 1%, which points to a synergistic interaction with additional environmental and genetic risk factors (Koizumi et al., 2016).

The gut microbiota is a crucial environmental component that affects the host’s metabolism and immunological homeostasis, according to growing evidence. The significance of the human gut microbiome in cardiac-cerebral vascular disorders such as high blood pressure, atherosclerosis, and stroke has gotten a lot of attention recently (Jie et al., 2017; Li et al., 2017; Peh et al., 2022). Additionally, according to a number of studies, changes in gut microbiome may precipitate in development of unruptured cerebral aneurysms (Li et al., 2020; Kawabata et al., 2022). Numerous metabolites produced by the gut microbiome in these disorders are reportedly taken into the circulatory system, where they are further processed by host enzymes, causing injury to the target organs (Koeth et al., 2013; Peh et al., 2022). Mineharu et al. collected feces from 27 MMD patients and 15 normal controls. They found no difference in α-diversity or β-diversity between patients with MMD and controls. Furthermore, their data showed that increased abundance of *Ruminococcus gnavus* and decreased abundance of *Roseburia inulinivorans* were associated with higher risk of MMD (Mineharu et al., 2022). However, the sample size they included was small.

Our hypothesis was that patients with MMD and HC have different types of microbiome profiles; the intima of the intracranial artery may change pathophysiologically and develop chronic inflammation as a result of this difference. We analyzed the gut microbiome of MMD patient and HC, and isolated the bacteria associated with MMD, in order to ascertain the relationship between MMD and the microbiome.

## Methods

### Standard protocol verification, registrations, and participant consents

Upon reasonable request, the corresponding author will provide the information supporting the outcomes of this manuscript. The Ethics Committee of Beijing Tiantan Hospital, Capital Medical University gave its authorization to the protocol prior to the case-control research starting (approval number KY2021-072-01). Each participant signed an informed consent. The investigation is reported in compliance with the STROBE declaration. The registration number of this study is NCT04890782.

### Study population

MMD was identified by digital subtraction angiography (DSA), based on Japanese recommendations released in 2012: (1) stenosis or blockage of the distal internal carotid and the proximal middle and anterior cerebral arteries and (2) unilateral or bilateral involvement (Fujimura et al., 2016). 72 adult patients (age ≥ 18 years) with MMD-type cerebrovascular disorder were prospectively enrolled between June 2021 and May 2022 from the Department of Neurosurgery, Beijing Tiantan Hospital, Capital Medical University. Among 72 adult patients, 10 patients with moyamoya syndrome and 2 with inadequate fecal sample were excluded (Scott and Smith, 2009; Kauv et al., 2019). Moyamoya syndrome refers to patients with MMD-type cerebrovascular disease accompanied by other basic diseases, such as arteriosclerosis, autoimmune disease, meningitis, and Down syndrome (Scott and Smith, 2009). In the end, 60 (83.33%) of the adult cases in the trial gave their permission. A control who had not undergo operations or taken any drugs was matched to every case with MMD. The HC were recruited from individuals who were shown not to have occlusive cerebrovascular disease by MRI and had regular medical examinations, and were matched according to their gender, age, and body mass index (BMI).

### Sample collection

After a 15-minute break spent sat, the patients’ right arms were tested for systolic blood pressure (SBP) and diastolic blood pressure (DBP) using a conventional mercury manometer. Weight (kg)/height (m^2^) was used to determine the BMI. After the participants had not eaten or drunk anything for 12 hours, venous blood samples were obtained. Fasting blood was examined for white blood cells (WBC), glucose, albumin (ALB), uric acid, triglyceride (TG), total cholesterol, high-density lipoprotein cholesterol, low-density lipoprotein cholesterol, and homocysteine (Hcy). Patients’ RNF213 p.R4810K variation was identified. The primers were generated in the following methodology. RNF213-4810F (rs112735431): 5’-GCCCTCCATTTCTAGCACAC-3’; and RNF21-4810R: 5’-AGCTGTGGCGAAAGCTTCTA-3’.

Data on the MMD patients’ age, sex, and comorbidities, such as hypertension, coronary artery disease, smoking, drinking, diabetes mellitus, thyroid disorders, and initial clinical features (ischemia and hemorrhage), were gathered at the time of admission. Two neurosurgeons utilized DSA to evaluate the Suzuki stage blindly.

For HC, fecal samples were collected at home at 06:30-08:30 am. The samples were stored at -80°C within 6 hours after production. For patients, fecal samples were collected at 06:30-08:30 am within 48 hours after admission. The samples were stored at -80°C within 2 hours after production. The online **Supplementary Methods** provide a description of the specifics.

### Bacterial DNA extraction and 16S rRNA sequencing

The CTAB technique was used to obtain microbial DNA from the stool specimens in accordance with the procedure. DNA samples that had been collected were amplified, DNA libraries had been created, and sequencing had been conducted using an Illumina NovaSeq platform. Amplified reads were analyzed. The online **Supplementary Methods** provide a description of the specifics.

### Microbiome bioinformatics

The generated FASTQ files were imported, demultiplexed, quality filtered, and analyzed utilising QIIME pipeline (Caporaso et al., 2010). Uparse program (Uparse v7.0.1001, http://drive5.com/uparse) was employed to analyze the sequences (Bokulich et al., 2013; Edgar, 2013). Sequences with ≥ 97% similarity were attributed to the same operational taxonomic units (OTUs). The Silva Database (http://www.arb-silva.de/) was employed depending on the Mothur algorithm to annotate taxonomic data for each sample sequence (Quast et al., 2013). All samples were included since rarefaction was conducted to a depth of 21,853 base pairs (100% of the minimum sample depth) to even the sequence depth. The online **Supplemental Methods** come with an explanation of the details.

In order to analyze the complexity of species diversity for a sample, three indices—Chao1, Shannon, and Simpson—are used. The R software, version 4.2.0, was used to generate and show each of these indices in our samples (Oksanen et al., 2017). The variations between specimens in terms of species complexity were assessed using β-diversity assessment. β-diversity on weighted unifrac estimated using the QIIME program (Version 1.9.1). Principal Coordinate Analysis (PCoA) was used to get principal coordinates and visualize from multidimensional data. Permutational multivariate ANOVA (PERMANOVA) was carried out to examine the statistical differences in β diversity across the cohorts, which was displayed by R software (Version 4.2.0) (McMurdie and Holmes, 2013). An envfit analysis associated to the PCoA was performed to identify whether sex, smoking, drinking, or hypertension have an effect on microbial composition distribution.

To identify substantially different taxa among the cohorts, the linear discriminant analysis effect size assessment was used (Segata et al., 2011). The linear discriminant analytic score was employed to evaluate the effect size of every distinctively abundant characteristic. When comparing the relative taxa abundances between the two groups, the threshold for a linear discriminant analytic score was established as ±2.0.

### Statistical analysis

The Student’s t test was employed to analyze continuous data reported as mean (standard deviation), and the Wilcoxon test was employed to analyze median with interquartile ranges. Chi-square test or Fisher’s exact analysis were used to assess categorical data, which were shown as percentages. To examine correlations between non-normal data, Spearman’s rank correlation was utilized. The Benjamin-Hochberg approach was employed to limit the false discovery rate when repeated comparisons were utilized to determine variations between 2 cohorts. By creating receiver-operating characteristic (ROC) curves and computing the area under the curve (AUC), the predictive performance of the MMD model was evaluated. For a more thorough investigation of the prediction performances, decision curve analysis (DCA) was employed. All analyses were two-sided, and statistical significance was set at *p* < 0.05. R program (version 4.2.0; https://www.r-project.org) and IBM SPSS Software (version 22.0; IBM Corp.) were employed for all statistical analyses.

Multivariate analysis by linear models (MaAsLin)2 is a tool to find associations between clinical metadata and bacterial abundance. We then correlated the microbiome data (generic level) with MMD via MaAsLin2 analysis adjusting for sex, age, smoking, drinking, and hypertension. All *p*-values were corrected for multiple testing using false discovery rate. False discovery rate adjusted *p* < 0.2 was considered statistically significant for taxonomic analysis.

## Results

### Patient’s characteristics

Sixty adult patients with MMD and 60 HC were consecutively recruited into our study. A total of 54 (45.00%) males and 66 (55.00%) females were enrolled, and the median age was 39.00 y (IQR, 31.00-46.50 y). **Table 1** illustrates a conclusion of the features of MMD patients and HC. The characteristics, including BMI, diabetes, hyperlipidemia, and thyroid disease did not show differences between patients and HC; however, subjects with MMD had greater rates of hypertension, smoking, and drinking compared with HC (*p* < 0.05 for all).

**Table 1:** Population characteristics among controls and MMD cases.

Characteristics Analyzed	MMD, n=60	HC, n=60	*P* value
Age, y	38.65 ± 9.43	38.93 ± 10.33	0.807
Men (%)	31 (51.67)	23 (38.33)	0.142
BMI, kg/m^2^	24.94 ± 4.16	23.88 ± 2.99	0.147
Hypertension	21 (35.00)	0 (0)	**0.000**
Diabetes	3 (5.00)	0 (0)	0.079
Hyperlipidemia	3 (5.00)	0 (0)	0.079
Thyroid disease	2 (3.33)	0 (0)	0.154
Smoking	14 (23.33)	1 (1.67)	**0.0003**
Drinking	8 (13.33)	0 (0)	**0.003**

MMD, moyamoya disease; HC, healthy controls; BMI, body mass index. The bold values means P < 0.05.

### Differences in gut microbiome between the MMD and HC cohorts

**Figure 1:**
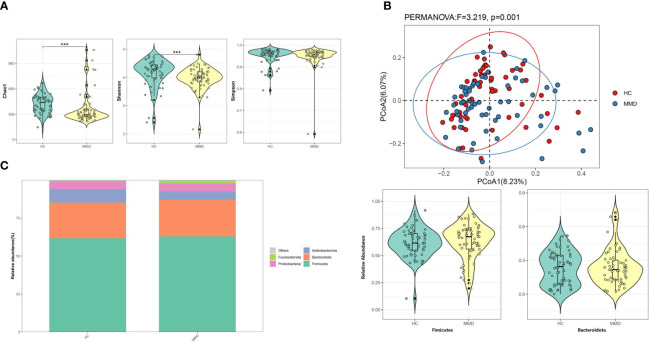
Comparison of microbial diversity and microbiome composition between MMD and HC groups. **(A)** The Chao 1 and the Shannon indices differed significantly between the two groups, while the Simpson index between the two groups did not show a difference. ***p < 0.001; **(B)** Principal coordinate analysis illustrating the grouping patterns of MMD and HC groups based on the weighted UniFrac distances. There was a significant difference in β-diversity (*r^2 = ^* 0.027, *p* = 0.001); **(C)** Distribution of the relative abundance of bacteria at the phylum level. The relative abundance of *Fimicutes* and *Bacteroidota* did not differ significantly between the groups. MMD, moyamoya disease; HC, healthy controls.

The 16,949 OTUs in the OUTs set of data for MMD individuals and HC were divided into 495 genera, 184 families, 108 orders, 47 classes, and 25 phyla. **Figure 1A** shows that although the Simpson index did not reveal a difference between the two cohorts, the Chao 1 and Shannon indices showed a substantial alteration (*p* < 0.001 for all). The weighted UniFrac distances between the two cohorts showed a substantial difference in β-diversity (*r^2 = ^* 0.027, *p* = 0.001, **Figure 1B**).

*Firmicutes* and *Bacteroidota* were the most prevalent phyla among all subjects, with 62.99 ± 16.05% and 24.00 ± 15.71 for the MMD cohort and 61.74 ± 13.11% and 23.39 ± 14.59% for the HC cohort, respectively (**Table 2** and **Figure 1C**). Additionally, there were no variations between patients and HC in terms of the relative abundance of *Firmicutes* and *Bacteroidota* (**Figure 1C**). At the phylum level, the relative abundance of *Fusobacteriota*/*Actinobacteria* was substantially higher/lower in the MMD group (false discovery rate, *q* = 0.049/0.011; **Table 2**).

**Table 2:** Relative abundance at the phylum level.

Relative abundance	MMD	HC	*P* value	*q* value
*Firmicutes*, %	62.99 ± 16.05	61.74 ± 13.11	0.239	1.000
*Bacteroidetes*, %	24.00 ± 15.71	23.39 ± 14.59	0.894	1.000
*Actinobacteria*, %	5.61 ± 5.76	8.91 ± 7.80	**0.005**	**0.049**
*Proteobacteria*, %	5.35 ± 11.09	5.27 ± 11.76	0.356	1.000
*Fusobacteriota*, %	1.51 ± 5.35	0.16 ± 0.83	**0.001**	**0.011**
*Verrucomicrobiota*, %	0.14 ± 0.41	0.24 ± 1.07	0.620	1.000
*Desulfobacteriota*, %	0.07 ± 0.09	0.16 ± 0.26	**0.028**	0.200
*Euryarchaeota*, %	0.01 ± 0.04	0.04 ± 0.19	0.718	1.000

MMD, moyamoya disease; HC, healthy controls. The bold values means P < 0.05.

**Figure 2:**
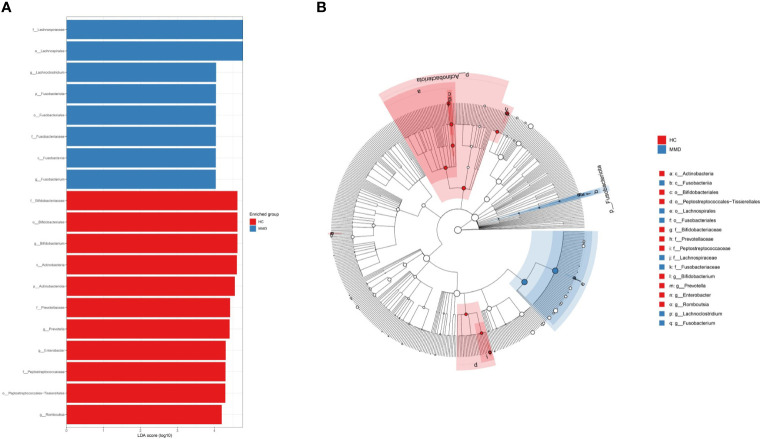
Discriminative taxa between MMD and HC groups. **(A)** Discriminative taxa between MMD and HC were determined using linear discriminant analysis effect size (LEfSe). The LEfSe analysis revealed that 2 bacterial phyla, 2 classes, 4 orders, 5 families, and 6 genera were significantly different between MMD and HC groups. The blue bar chart represents the bacteria that were more abundant in the MMD group and the red bar chart represents the bacteria that were more abundant in the HC group; **(B)** The cladograms report the taxa showing different abundance values according to LEfSe. The 2 genera *Lachnoclostridium* and *Fusobacterium* had higher abundance in the MMD group as compared to those in the HC group; The 4 genera *Bifidobacterium*, *Prevotella*, *Enterobacter*, and *Romboutsia* had higher abundance in the HC group as compared to those in the MMD group. MMD, moyamoya disease; HC, healthy controls.

**Figure 3:**
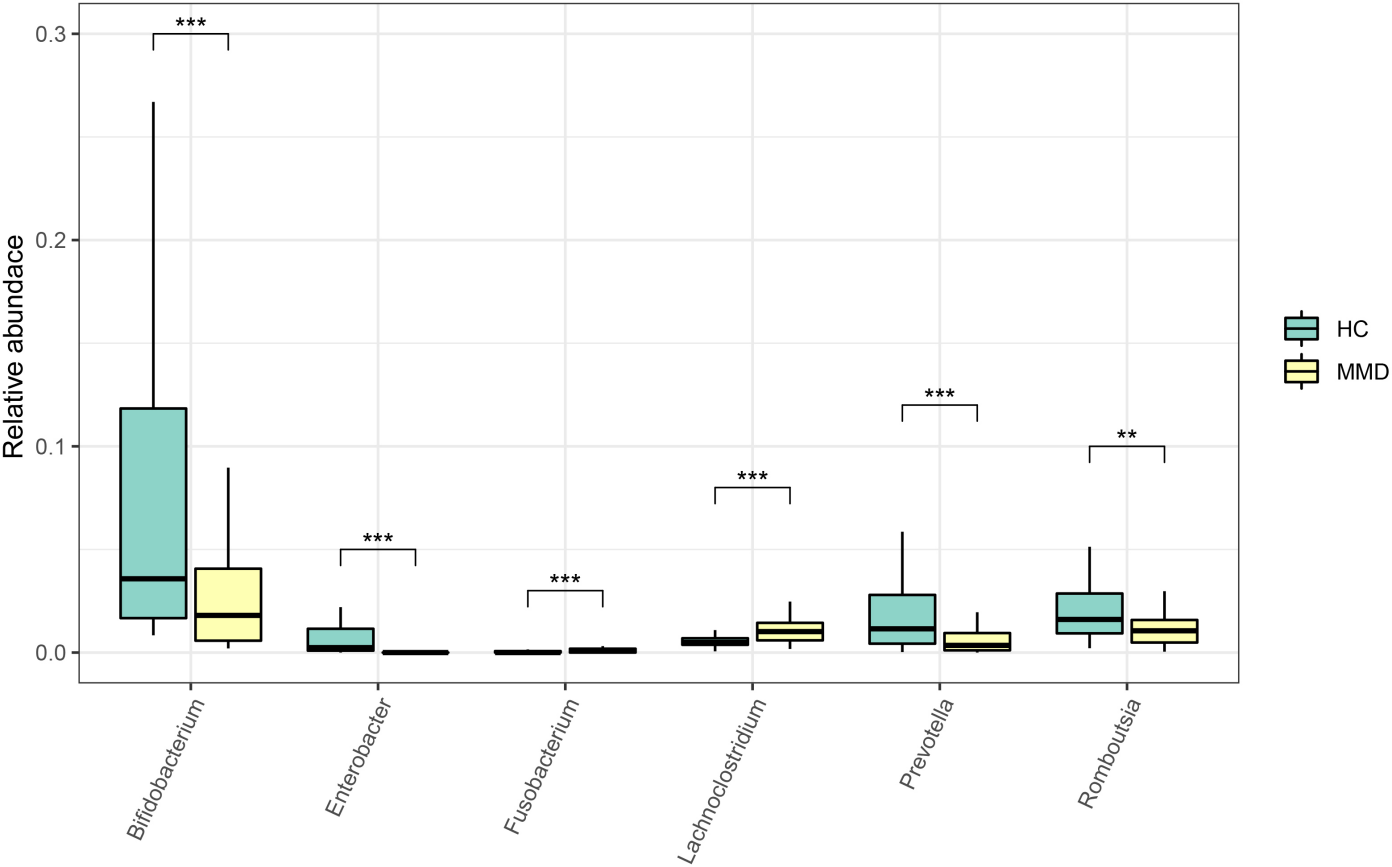
Comparison of the relative abundance of the differential genera between MMD and HC groups. ***p < 0.001; **0.001 < *p* < 0.01. MMD, moyamoya disease; HC, healthy controls.

**Figure 4:**
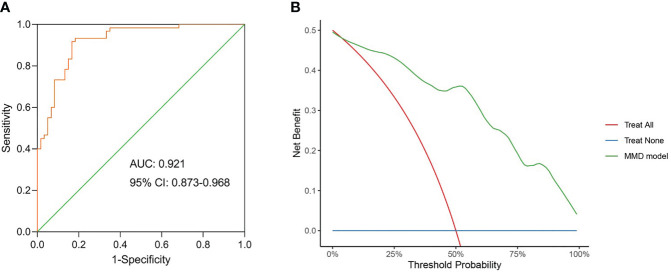
The model constructed using the differential genera can be used as a disease classifier to differentiate MMD patients from HC. **(A)** Receiver-operating characteristic (ROC) of the model constructed using the differential genera. The diagonal line in the graph marks an area under the curve (AUC) of 0.5. The AUC value was 0.921 with 95% confidence intervals (CI) of 0.873 to 0.968 (sensitivity 91.7%; specificity 83.3%; Youden index 0.750); **(B)** Decision curve analysis (DCA) for the model constructed using the differential genera. DCA showed wide and practical range of threshold probabilities for MMD. MMD, moyamoya disease; HC, healthy controls.

In the linear discriminant analysis effect size analysis, we identified 2 bacterial phyla, 2 classes, 4 orders, 5 families, and 6 genera that varied noticeably in their relative abundance in the MMD and HC cohorts (**Figure 2A**). For the examination of the abundance ratio of these 6 genera, 2 genera were enriched in the MMD cohort; 4 genera were enriched in the HC cohort (**Figure 2B**). The 2 genera *Lachnoclostridium* and *Fusobacterium* were more prevalent in the MMD cohort than the HC cohort (*p* < 0.001 for all, **Figure 3**). The 4 genera *Bifidobacterium*, *Prevotella*, *Enterobacter*, and *Romboutsia* were more abundant in the HC cohort as compared to those in the MMD cohort (*p* < 0.001 for *Bifidobacterium*, *Prevotella*, and *Enterobacter*; *p* < 0.01 for *Romboutsia*, **Figure 3**). Using MaAsLin2, we were able to identify differences in taxa abundances between MMD and HC while correcting for sex, age, smoking, drinking, and hypertension. By MaAsLin2 analysis, MMD was associated with decreased relative abundances of *Enterobacter* and *Bifidobacterium* and increased relative abundances of *Lachnoclostridium* and *Fusobacterium* (**Table 3**, **Figure VI**). We created a prediction model employing these 4 genera as a disease classifier after identifying them. The AUC value was 0.921 with 95% confidence interval of 0.873 to 0.968 between MMD and HC groups (sensitivity 91.7%; specificity 83.3%; Youden index 0.750) (**Figure 4A**). Due to the wide and practical ranges of the threshold probabilities, the outcomes of DCA supported and verified the utilization of these 4 genera in the anticipation of MMD (**Figure 4B**). There was no difference in the detection rate of *Enterobacter* and *Fusobacterium* between males and females (**Figure II** in the **Supplementary materials**). In addition, a higher proportion of sex (envfit analysis, *r^2 = ^* 0.020, *p* = 0.328), smoking (envfit analysis, *r^2 = ^* 0.018, *p* = 0.358), drinking (envfit analysis, *r^2 = ^* 0.000, *p* = 0.995), and hypertension (envfit analysis, *r^2 = ^* 0.035, *p* = 0.102), in patients with MMD did not affect the difference of microbiome between patients and controls (**Figure III** in the **Supplementary materials**).

**Table 3:** Differential bacteria genera between patients with MMD and HC.

Genera	MMD	HC	coef	*P* value	*q* value
*Bifidobacterium*, %	3.25 ± 0.53	7.26 ± 0.96	-0.390	**0.027**	**0.123**
*Enterobacter*, %	0.03 ± 0.02	2.30 ± 1.32	-2.339	**0.000**	**0.000**
*Fusobacterium*, %	1.49 ± 0.68	0.16 ± 0.11	1.517	**0.008**	**0.063**
*Lachnoclostridium*, %	1.68 ± 0.36	0.59 ± 0.05	0.931	**0.000**	**0.000**

MMD, moyamoya disease; HC, healthy controls. The differential genera between MMD patients and HC were determined by MaAsLin2, adjusting for age, sex, smoking, drinking, and hypertension. Only the genera with q value < 0.2 were shown. The bold values means P < 0.05.

### The relationship between alterations of the gut microbiota and medical indices in subjects with MMD

As shown in **Figure I** in the Supplementary materials, the 4 genera were significantly correlated with 4 clinical indices (*p* < 0.05 for all). The abundance of *Enterobacter* was positively correlated with serum TG levels. Furthermore, the abundance of *Bifidobacterium* and *Lachnoclostridium* were positively correlated with WBC and SBP, respectively. The abundance of *Fusobacterium* was negatively correlated with BMI.

### Differences in gut microbiota among different MMD subtypes

**Figure 5:**
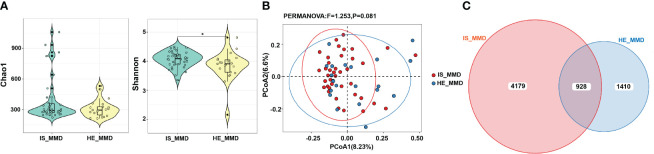
Comparison of microbial diversity between ischemic (IS) and hemorrhagic (HE) MMD. **(A)** The Shannon index differed between the two groups; **(B)** There was no significant difference in β-diversity (*p* > 0.05); **(C)** The Venn diagram displaying the overlaps between these two groups indicated that 928 OTUs were shared among the two groups. *0.01 < *p* < 0.05. MMD, moyamoya disease; IS_MMD, ischemic moyamoya disease; HE_MMD, hemorrhagic moyamoya disease.

The Shannon index differed substantially between the ischemic and hemorrhagic cohorts (*p* < 0.05, **Figure 5A**). However, no substantial alterations were detected in the weighted UniFrac distances between these both cohorts in β-diversity (**Figure 5B**). The Venn figure presenting the overlaps among these two cohorts indicated that 928 OTUs were mutual between the two cohorts (**Figure 5C**). In the linear discriminant analysis effect size analysis, we did not recognize that the relative abundance of any phyla, classes, orders, families, and genera differed significantly between the ischemic and hemorrhagic groups. As demonstrated in **Figure IV, V** in the **Supplementary materials**, there was also no alteration in β-diversity among MMD patients with wild-type p.R4810K variants (GG)/Suzuki stage of 0-2 and heterozygous p.R4810K variants (GA)/Suzuki stage of 3-6. Furthermore, we did not identify that the relative abundance of any phyla, classes, orders, families, and genera that differed significantly between these groups in the linear discriminant analysis effect size analysis.

## Discussion

In this study, at the phylum level, the relative abundance of *Fusobacteriota*/*Actinobacteria* was considerably higher/lower in the MMD cohort than that in the HC cohort. In the linear discriminant analysis effect size analysis, 2 bacterial phyla, 2 classes, 4 orders, 5 families, and 6 genera were detected. We discovered that the abundance of the 2 genera, *Lachnoclostridium* and *Fusobacterium*, rose in the MMD group, while the abundance of the 2 genera, *Bifidobacterium* and *Enterobacter* diminished in the MMD group. Additionally, we created a disease classification model employing these 4 markers (genera), and it was shown to be beneficial for medical prediction.

The Chao 1, Shannon, and Simpson indices are frequently employed to estimate the diversity within a single environment or sample (α-diversity). Rare species are given greater weight by the Chao 1 and Shannon indices than by the Simpson index, which favors common species (Qian et al., 2020). The Simpson index did not vary substantially between the two cohort in this study, but the Chao 1 and Shannon indices did, suggesting that the rare species may have a stronger impact on the differences than the common species. β-diversity index varied among the two cohorts, indicating that the gut microbiota of the two groups was distinct.

The results of a diversity study showed that the microbiome structure of both cohorts differed. Univariate community assessment further emphasized the variations in the both cohorts’ microbiome. *Fusobacterium* is an opportunistic commensal anaerobe in the oral cavity or colon (Brennan and Garrett, 2019). In recent years, many investigations have exhibited that *Fusobacterium* is involved in the growth and progression of colorectal cancer by regulating immune inflammatory factors (Kostic et al., 2013; Rubinstein et al., 2013). In addition, *Fusobacterium* can promote the production of pro-inflammatory factors and reactive oxygen species, which may play a key role in chronic inflammation (Kang et al., 2019). In this investigation, the abundance of *Fusobacterium* was higher in the MMD group than in the HC group. Therefore, it is reasonable to speculate that chronic inflammation mediated by *Fusobacterium* may have a potential relationship with the development of MMD. By digesting type IV collagen, matrix metalloproteinase (MMP)-9 destroys endothelium basal lamina and tight junction proteins, and its over activation causes endothelial instability (Murphy and Nagase, 2008). The serum MMP-9 may act as a biomarker for bleeding anticipation in MMD, according to a prospective study that used plasma samples from 84 subjects with MMD (Lu et al., 2021). It has been shown that *Fusobacterium* can induce the production of MMP-9 (Suzuki et al., 2022). So, it is also possible that *Fusobacterium* mediates the pathogenesis of MMD by inducing the production of MMP-9. More in-depth studies are needed to confirm the above ideas.

The extremely polyphyletic class Clostridia includes the recently founded genus *Lachnoclostridium* (Yutin and Galperin, 2013). Additionally, compared to normal controls, colorectal cancer patients had a greater relative abundance of *Lachnoclostridium* (Liang et al., 2020). Wang et al. firstly suggested a dose-dependent relationship between plasma levels of trimethylamine-N-oxide (TMAO) and risk of cardiovascular disorders in individuals with heart problems in 2011 (Wang et al., 2011). Furthermore, the levels of TMAO were positively correlated with the prevalence of hypertension or hyperhomocysteinemia (Nie et al., 2018; Ge et al., 2020b). Trimethylamine (TMA), a byproduct of intestinal microbial metabolism, is converted to TMAO in the liver via flavin-containing monooxygenase 3 (Bennett et al., 2013). A recent study demonstrated that *Lachnoclostridium* can enhance the synthesis of TMA and so promote atherosclerosis progression (Cai et al., 2022). Previous research has shown that elevated Hcy was connected to a greater risk of MMD (Ge et al., 2020a). In this investigation, the abundance of *Lachnoclostridium* was higher in the MMD cohort than in the HC cohort. Therefore, we hypothesized that *Lachnoclostridium* may influence the levels of Hcy and thus involve with the onset of MMD by promoting the production of TMAO. What’s more interesting is that among individuals with MMD, we discovered a positive association between the relative abundance of *Lachnoclostridium* and SBP. This can also be explained by the effect of *Lachnoclostridium* on TMAO synthesis. The relative abundance of *Lachnoclostridium* might be predictive of blood pressure in patients with MMD.

*Bifidobacterium* is an important intestinal beneficial microorganism. Previous research has already indicated that *Bifidobacterium* was negatively correlated with plasma TMAO and TMA (Chen et al., 2016). In this experiment, the MMD cohort had a lesser abundance of *Bifidobacterium* than the HC cohort. As mentioned earlier, the emergence of this trend was logical. *Enterobacter* may exert a probiotic effect in the gastrointestinal tract of humans (Robinson, 2014). The relative abundance of *Enterobacter* in MMD patients were also reduced in this study. *Bifidobacterium* and *Enterobacter* may have antagonistic effect on the occurrence or development of MMD.

Mineharu et al. collected feces from 27 MMD patients and 15 normal controls. They found no difference in α-diversity or β-diversity between patients with MMD and controls (Mineharu et al., 2022). We speculated that the reason their findings were not consistent with ours is because of their small sample size. Furthermore, they found that increased abundance of *Ruminococcus gnavus* and *Peptostreptococcaceae* and decreased abundance of *Roseburia inulinivorans* in gut microbiota in MMD patients. However, our data yielded the opposite conclusion. The present study demonstrated an increase in the abundance of *Peptostreptococcaceae* in healthy controls. In addition, although we found a higher abundance of *Actinobacteria* in the HC group, we did not find a difference in the abundance of *Roseburia* between the two groups. Although species level was not involved in this study, there were significant differences between our study and the previous study. We speculated that gut microbiota in population may also change depending on geographic location. Therefore, international multicenter replication studies are needed to confirm the results of the present study.

The 4 genera found in our investigation were employed to create a predictive model. We utilized ROC and DCA to assess the predictive power of the model. The AUC value of the model was. The results of DCA also indicated that the model has good predictive power. It’s still not apparent if adding the clinical characteristics will help the prediction model get any better. Nevertheless, for the diagnosis of MMD, DSA is still necessary. The results of this study may provide some clues to the etiology of MMD. Moreover, we did not observe differences in gut microbiota between subgroups of MMD. Therefore, we hypothesized that similar alterations in gut microbiota may lead to different phenotypes of MMD. RNF213 p.R4810K variant did not appear to affect the gut microbiota of MMD patients. The underlying mechanisms of these hypotheses need to be confirmed by further studies.

This study has a number of restrictions. First, metabolomics and heredity as well as other risk variables were not studied. Second, these findings could not be extended to children or other races, as only Chinese adult patients with MMD were included. Third, to ascertain the cause-and-effect connection between the gut microbiome and the onset of MMD, more research, including longitudinal human studies, is required. Fourth, unequal distribution of clinical variables (smoking, drinking, use of medication, etc) between cases and controls may affect the distribution of gut microbiome to some extent. Finally, this is a prospective study using clinical information from a single institute. The results’ applicability is constrained, necessitating additional validation in a different cohort or location.

## Conclusions

In conclusion, patients with MMD had changed gut microbiota, which were distinguished by elevated abundance of *Lachnoclostridium* and *Fusobacterium* and reduced abundance of *Bifidobacterium* and *Enterobacter*. Additionally, these 4 genera utilized in the model’s construction were able to accurately identify whether a person had MMD or not, indicating that these genera may be applied in clinical settings as predictors and biomarkers.

## Data availability statement

The datasets presented in this study can be found in online repositories. The names of the repository/repositories and accession number(s) can be found in the article/**Supplementary Material**.

## Ethics statement

The studies involving humans were approved by The Ethics Committee of Beijing Tiantan Hospital, Capital Medical University. The studies were conducted in accordance with the local legislation and institutional requirements. The participants provided their written informed consent to participate in this study.

## Author contributions

Conceptualization and methodology: PG and DZ. Data curation and writing original draft: XiY. Visualization and investigation: YuZ and WL. Supervision: QZ, XuY, and JZ. Software and validation: XL, RW, and YaZ. All authors contributed to the article and approved the submitted version.
