# Supplementary material for: Gut microbiota in adults with moyamoya disease: characteristics and biomarker identification
Source: Front Cell Infect Microbiol. 2023 Oct 17;13:1252681. doi: 10.3389/fcimb.2023.1252681 (PMC10616959; doi:10.3389/fcimb.2023.1252681)
Supplement: Supplementary file 1 [file DataSheet_1.pdf]

Supplementary Materials

Supplementary Methods

Fecal sample collection

Each individual provided a fresh fecal sample at 06:30-08:30 am; this was delivered immediately to the laboratory in a gel pack within insulated containers and shipped at -20°C. In the laboratory, the sample was divided into five aliquots of 200 mg and immediately stored at -80°C. The samples from healthy controls and MMD patients were stored at -80°C within 6 hours and 2 hours after production, respectively. The sample that stayed in room temperature more than 2 hours was discarded.

Bacterial DNA extraction and 16S rRNA sequencing

Total genome DNA from samples was extracted using CTAB method. DNA concentration and purity was monitored on 1% agarose gels. According to the concentrations, DNA was diluted to 1ng/µL using sterile water.

16S rRNA/18S rRNA/ITS genes of distinct regions (16S V4/16S V3/16S V3-V4/16S V4-V5, 18S V4/18S V9, ITS1/ITS2, Arc V4) were amplified used specific primer (e.g. 16S V4: 515F-806R, 18S V4: 528F-706R, 18S V9:1380F-1510R, et. al) with the barcode. All PCR reactions were carried out with 15 µL of Phusion High-Fidelity PCR Master Mix (New England Biolabs); 2 µM of forward and reverse primers, and about 10 ng template DNA. Thermal cycling consisted of initial denaturation at 98 °C for 1 min, followed by 30 cycles of denaturation at 98 °C for 10 s, annealing at 50 °C for 30 s, and elongation at 72 °C for 30 s. Finally, 72 °C for 5 min.

Mix same volume of 1X loading buffer (contained SYB green) with PCR products and operate electrophoresis on 2% agarose gel for detection. PCR products was mixed in equidensity ratios. Then, mixture PCR products was purified with Qiagen Gel Extraction Kit (Qiagen, Germany).

Sequencing libraries were generated using TruSeq DNA PCR-Free Sample Preparation Kit (Illumina, USA) following manufacture's recommendations and index codes were added. The library quality was assessed on the Qubit@ 2.0 Fluorometer

(Thermo Scientific) and Agilent Bioanalyzer 2100 system. At last, the library was sequenced on an Illumina NovaSeq platform and 250 bp paired-end were generated.

Microbiome Bioinformatics

Paired-end reads was assigned to samples based on their unique barcode and truncated by cutting off the barcode and primer sequence. Paired-end reads were merged using FLASH (V1.2.7, http://ccb.jhu.edu/software/FLASH), a very fast and accurate analysis tool, which was designed to merge paired-end reads when at least some of the reads overlap the read generated from the opposite end of the same DNA fragment, and the splicing sequences were called raw tags [1]. Quality filtering on the raw tags were performed under specific filtering conditions to obtain the high-quality clean tags according to the QIIME (V1.9.1, http://qiime.org/scripts/split_libraries_fastq.html) quality controlled process [2, 3]. The tags were compared with the reference database (Sliva database, http://www.arb-silva.de/) using UCHIME algorithm (UCHIME, http://www.drive5.com/usearch/manual/uchime_algo.html) to detect chimera sequences, and then the chimera sequences were removed [4, 5]. Then the Effective Tags finally obtained.

Sequences analysis were performed by Uparse software (Uparse v7.0.1001, http://drive5.com/uparse) [6]. Sequences with ≥97% similarity were assigned to the same OTUs. Representative sequence for each OUT was screened for further annotation. For each representative sequence, the Silva Database (http://www.arb-silva.de/) was used based on Mothur algorithm to annotate taxonomic information [7]. In order to study phylogenetic relationship of different OTUs, and the difference of the dominant species in different samples (groups), multiple sequence alignment were conducted using the MUSCLE software (Version 3.8.31, http://www.drive5.com/muscle/) [8]. OTUs abundance information were normalized using a standard of sequence number corresponding to the sample with the least sequences. Subsequent analysis of alpha diversity and beta diversity were all performed basing on this output normalized data.

Reference

[1] Magoč T, Salzberg SL. FLASH: fast length adjustment of short reads to improve genome assemblies. Bioinformatics. 2011;27:2957-63.

[2] Bokulich NA, Subramanian S, Faith JJ, Gevers D, Gordon JI, Knight R, et al. Quality-filtering vastly improves diversity estimates from Illumina amplicon sequencing. Nat Methods. 2013;10:57-9.

[3] Caporaso JG, Kuczynski J, Stombaugh J, Bittinger K, Bushman FD, Costello EK, et al. QIIME allows analysis of high-throughput community sequencing data. Nat Methods. 2010;7:335-6.

[4] Haas BJ, Gevers D, Earl AM, Feldgarden M, Ward DV, Giannoukos G, et al. Chimeric 16S rRNA sequence formation and detection in Sanger and 454-pyrosequenced PCR amplicons. Genome Res. 2011;21:494-504.

[5] Edgar RC, Haas BJ, Clemente JC, Quince C, Knight R. UCHIME improves sensitivity and speed of chimera detection. Bioinformatics. 2011;27:2194-200.

[6] Edgar RC. UPARSE: highly accurate OTU sequences from microbial amplicon reads. Nat Methods. 2013;10:996-8.

[7] Quast C, Pruesse E, Yilmaz P, Gerken J, Schweer T, Yarza P, et al. The SILVA ribosomal RNA gene database project: improved data processing and web-based tools. Nucleic Acids Res. 2013;41:D590-6.

[8] Edgar RC. MUSCLE: multiple sequence alignment with high accuracy and high throughput. Nucleic Acids Res. 2004;32:1792-7.

Table I. Comparison of baseline characteristics between ischemic and hemorrhagic MMD

Characteristics	Ischemic MMD, n=39	Hemorrhagic MMD, n=21	*P*-value
Age, y	37.92±8.69	40.00±10.77	0.420
Men (%)	21 (53.85)	10 (47.62)	0.645
BMI, kg/m^2	25.15±3.52	24.55±5.22	0.593
Hypertension	16 (41.03)	5 (23.81)	0.182
Diabetes	2 (5.13)	1 (4.76)	0.951
Hyperlipidemia	3 (7.69)	0 (0)	0.192
Thyroid disease	1 (2.56)	1 (4.76)	0.651
Smoking	8 (20.51)	6 (28.57)	0.482
Drinking	5 (12.82)	3 (14.29)	0.874
Suzuki stage			0.679
0-2	11 (28.21)	7 (33.33)	
3-6	28 (71.79)	14 (66.67)	

MMD, moyamoya disease; BMI, body mass index

Table II. Comparison of baseline characteristics between GG and GA MMD

Characteristics	GG MMD, n=43	GA MMD, n=17	*P*-value
Age, y	39.74±9.49	35.88±8.96	0.155
Men (%)	24 (55.81)	7 (41.18)	0.307
BMI, kg/m^2	25.37±4.42	23.85±3.26	0.204
Hypertension	15 (34.88)	6 (35.29)	0.976
Diabetes	3 (6.98)	0 (0)	0.264
Hyperlipidemia	3 (6.98)	0 (0)	0.264
Thyroid disease	2 (4.65)	0 (0)	0.366
Smoking	11 (25.58)	3 (17.65)	0.513
Drinking	6 (13.95)	2 (11.76)	0.822
Suzuki stage			0.053
0-2	16 (37.21)	2 (11.76)	
3-6	27 (62.79)	15 (88.24)	

MMD, moyamoya disease; BMI, body mass index; GG, wild-type p.R4810K variants; GA, heterozygous p.R4810K variants

Table III. Comparison of baseline characteristics between MMD patients in Suzuki stage 0-2 and 3-6

Characteristics	Suzuki stage 0-2, n=18	Suzuki stage 3-6, n=42	*P*-value
Age, y	39.17±9.12	38.43±9.66	0.784
Men (%)	10 (55.55)	21 (50.00)	0.693
BMI, kg/m^2	26.92±5.14	24.09±3.39	**0.014**
Hypertension	5 (27.78)	16 (38.10)	0.443
Diabetes	2 (11.11)	1 (2.38)	0.155
Hyperlipidemia	1 (5.55)	2 (4.76)	0.897
Thyroid disease	1 (5.55)	1 (2.38)	0.530
Smoking	6 (33.33)	8 (19.05)	0.231
Drinking	3 (16.67)	5 (11.90)	0.619

MMD, moyamoya disease; BMI, body mass index

Table IV. Comparison of drugs between MMD and HC group

Drugs	MMD, n=60	HC, n=60	*P*-value
Aspirin	2 (3.33)	0 (0)	0.154
Metformin	3 (5.00)	0 (0)	0.079
Statin	3 (5.00)	0 (0)	0.079
CCB	12 (20.00)	0 (0)	**0.0003**
ACEI	1 (1.67)	0 (0)	0.315
ARB	3 (5.00)	0 (0)	0.079

MMD, moyamoya disease; HC, healthy controls; CCB, calcium channel blocker; ACEI, angiotensin converting enzyme inhibitor; ABR, angiotensin 2 receptor blocker

Table V. The relationship between drugs and Genus *Fusobacterium* by 16S rRNA

Drugs	*Fusobacterium* (-), n=56	*Fusobacterium* (+), n=64	*P*-value
Aspirin	2 (3.57)	0 (0)	0.127
Metformin	1 (1.79)	2 (3.13)	0.639
Statin	2 (3.57)	1 (1.56)	0.482
CCB	6 (10.71)	6 (9.38)	0.807
ACEI	0 (0)	1 (1.56)	0.939
ARB	0 (0)	3 (4.69)	0.101

CCB, calcium channel blocker; ACEI, angiotensin converting enzyme inhibitor; ABR, angiotensin 2 receptor blocker

Table VI. The relationship between drugs and Genus *Enterobacter* by 16S rRNA

Drugs	*Enterobacter* (-), n=55	*Enterobacter* (+), n=65	*P*-value
Aspirin	1 (1.82)	1 (1.54)	0.905
Metformin	2 (3.64)	1 (1.54)	0.463
Statin	2 (3.64)	1 (1.54)	0.463
CCB	10 (18.18)	2 (3.08)	**0.006**
ACEI	1 (1.82)	0 (0)	0.275
ARB	1 (1.82)	2 (3.08)	0.660

CCB, calcium channel blocker; ACEI, angiotensin converting enzyme inhibitor; ABR, angiotensin 2 receptor blocker

Table VII. Univariate analysis for each bacterial genus

Genera	MMD	HC	coef	*P* value	*q* value
Bifidobacterium, %	3.25±0.53	7.26±0.96	-0.249	0.086	0.104
Enterobacter, %	0.03±0.02	2.30±1.32	-2.332	**0.000**	**0.000**
Fusobacterium, %	1.49±0.68	0.16±0.11	1.205	**0.000**	**0.000**
Lachnoclostridium, %	1.68±0.36	0.59±0.05	0.926	**0.000**	**0.000**
Prevotella, %	2.63±0.87	4.81±1.10	-0.568	**0.020**	**0.029**
Romboutsia, %	1.68±0.35	3.44±0.92	-0.062	0.701	0.701

Figure I. Relationship between the relative abundance of the differential genera and clinical indices.

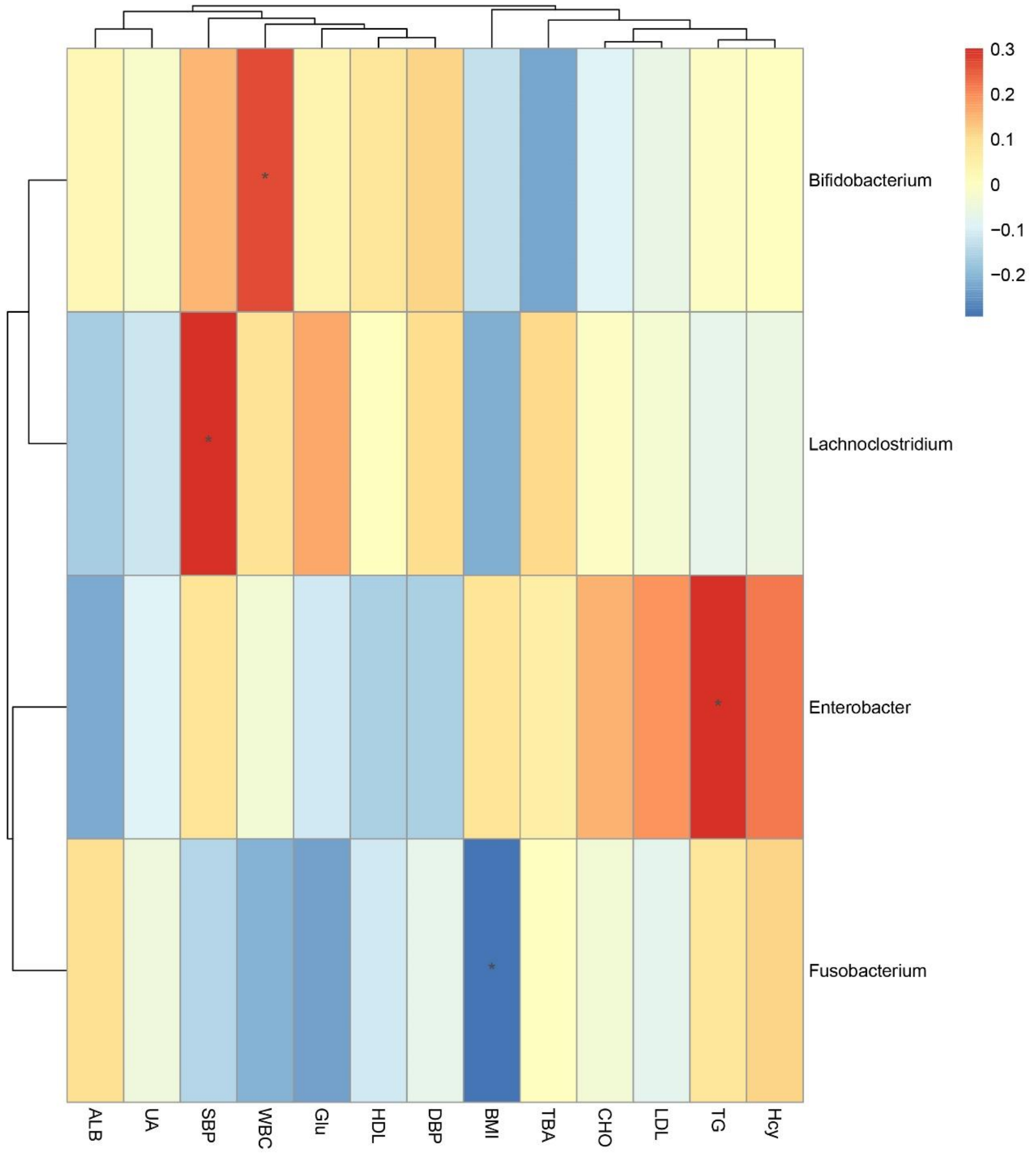

The relative abundance of *Enterobacter* and *Prevotella* were positively correlated with serum TG levels; The relative abundance of *Bifidobacterium* and *Lachnoclostridium* were positively correlated with WBC and SBP, respectively; The relative abundance of *Fusobacterium* were negatively correlated with BMI. *0.01 < p < 0.05; **0.001 < p < 0.01. ALB, albumin; SBP, systolic blood pressure; WBC, white blood cells; Glu, glucose; HDL, high-density lipoprotein; DBP, diastolic blood pressure; TBA, total bile acid; UA, uric acid; BMI, body mass index; TG, triglyceride; Hcy, homocysteine; CHO, total cholesterol; LDL, low-density lipoprotein

Figure II. Comparison of the existence of genus *Enterobacter* and *Fusobacterium* between males and females.

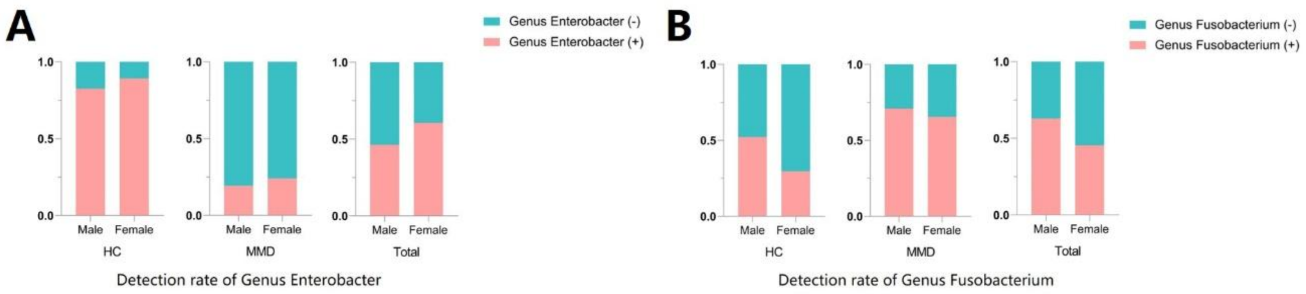

A, In the HC group, Genus *Enterobacter* was identified in 82.61% of males and 89.19% of females ($p = 0.446$). In the MMD group, Genus *Enterobacter* was identified in 19.35% of males and 24.14% of females ($p = 0.653$). In total, *Enterobacter* was identified in 46.30% of males and 60.61% of females ($p = 0.118$); **B**, In the HC group, Genus *Fusobacterium* was identified in 52.17% of males and 29.73% of females ($p = 0.082$). In the MMD group, Genus *Fusobacterium* was identified in 70.97% of males and 65.52% of females ($p = 0.650$). In total, *Fusobacterium* was identified in 62.96% of males and 45.45% of females ($p = 0.056$).

Figure III. Envfit analysis associated to the PCoA was performed to identify whether sex, smoking, drinking, or hypertension have an effect on microbial composition distribution.

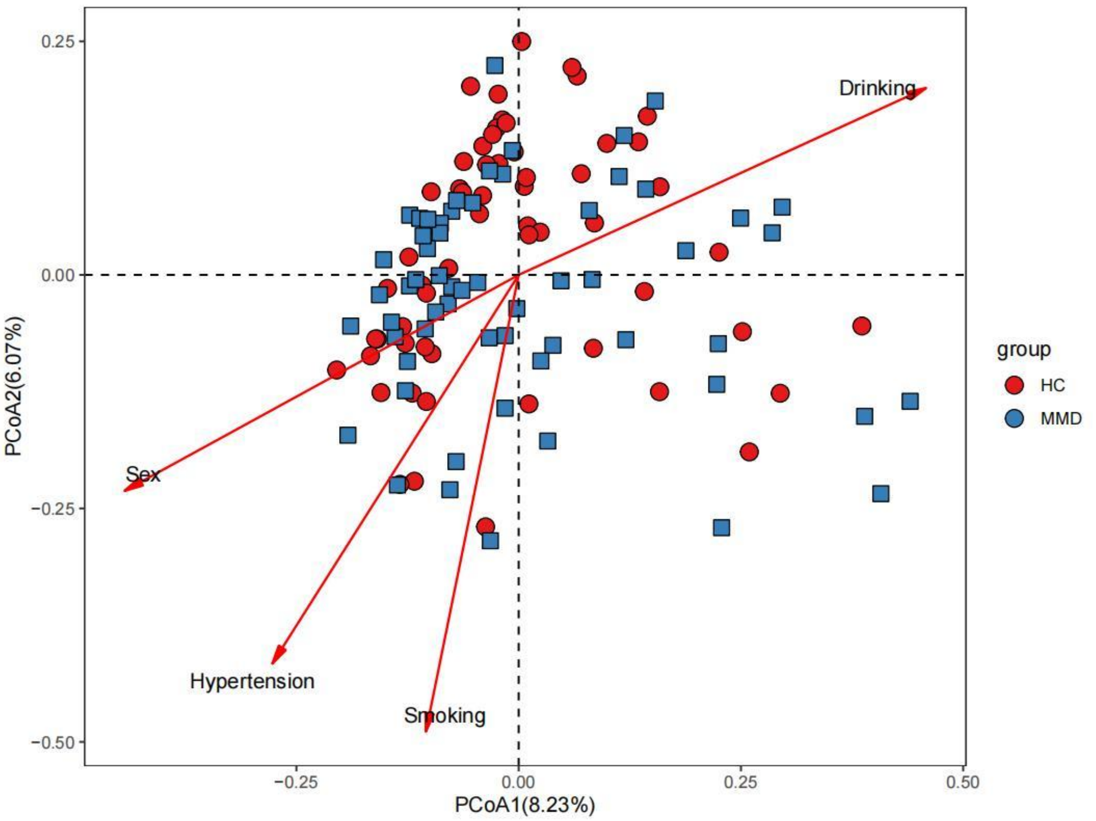

A higher proportion of sex (envfit analysis, $r^2 = 0.020$, $p = 0.328$), smoking (envfit analysis, $r^2 = 0.018$, $p = 0.358$), drinking (envfit analysis, $r^2 = 0.000$, $p = 0.995$), and hypertension (envfit analysis, $r^2 = 0.035$, $p = 0.102$), in patients with MMD did not affect the difference of microbiome between patients and controls.

Figure IV. Comparison of microbial diversity between MMD with wild-type p.R4810K variants (GG) and heterozygous p.R4810K variants (GA).

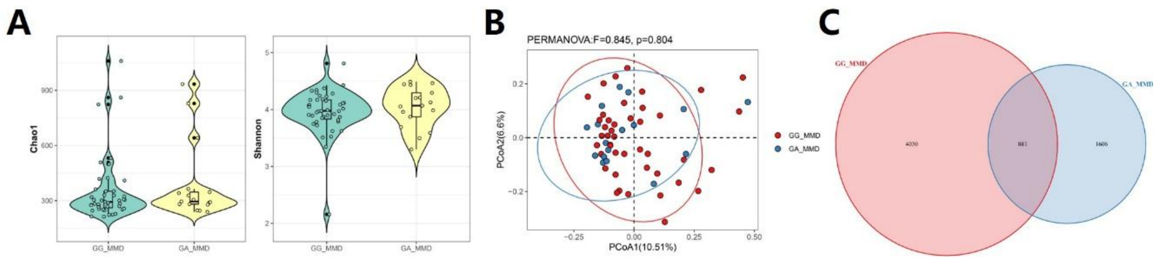

A, There was no significant difference in α-diversity ($p > 0.05$); **B**, There was no significant difference in β-diversity ($p > 0.05$); **C**, The Venn diagram displaying the overlaps between these two groups indicated that 881 OTUs were shared among the two groups. MMD,

moyamoya disease; GG_MMD, moyamoya disease with wild-type p.R4810K variants; GA_MMD, moyamoya disease with heterozygous p.R4810K variants

Figure V. Comparison of microbial diversity between MMD with Suzuki stage of 0-2 and Suzuki stage of 3-6.

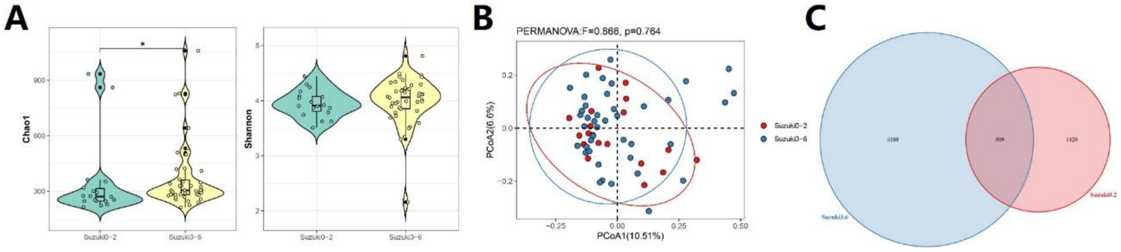

A, The Chao 1 index differed between the two groups; **B**, There was no significant difference in β-diversity ($p > 0.05$); **C**, The Venn diagram displaying the overlaps between these two groups indicated that 909 OTUs were shared among the two groups. MMD, moyamoya disease. *$p < 0.05$.

Figure VI. Genera association with population characteristics. Correlation and statistical significance were determined by MaAsLin2 with multiple comparison adjustment by false discovery rate.

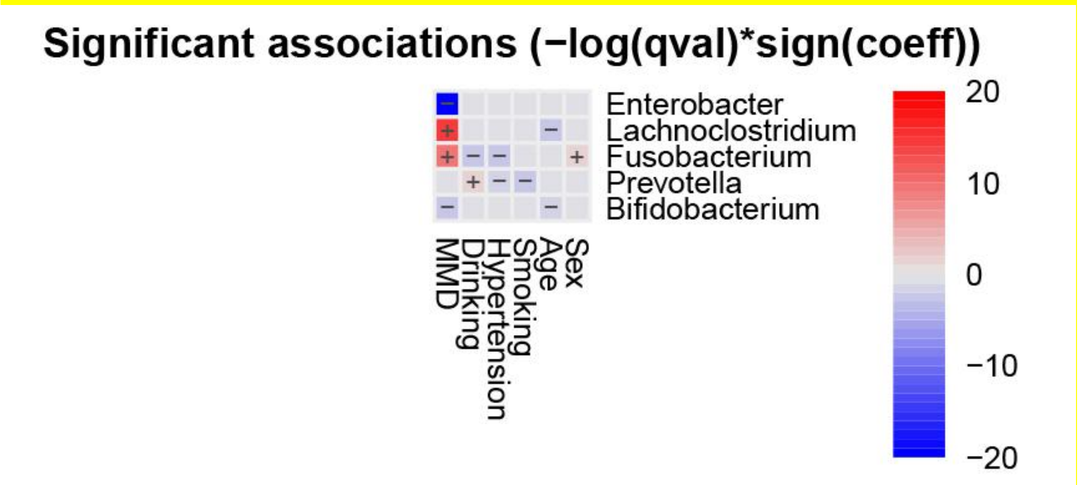

MMD was associated with decreased relative abundances of *Enterobacter* and *Bifidobacterium* and increased relative abundances of *Lachnoclostridium* and *Fusobacterium*. False discovery rate adjusted $p < 0.2$ was considered statistically significant for taxonomic analysis.
